# Alcohol consumption and sport: a cross-sectional study of alcohol management practices associated with at-risk alcohol consumption at community football clubs

**DOI:** 10.1186/1471-2458-13-762

**Published:** 2013-08-16

**Authors:** Melanie Kingsland, Luke Wolfenden, Bosco C Rowland, Karen E Gillham, Vanessa J Kennedy, Robyn L Ramsden, Richard W Colbran, Sarah Weir, John H Wiggers

**Affiliations:** 1 The University of Newcastle, School of Medicine and Public Health, Callaghan, New South Wales 2308, Australia; 2 Hunter New England Population Health, Locked Bag 10, Wallsend, New South Wales 2287, Australia; 3 NSW Cancer Institute, Australian Technology Park, Level 9, 8 Central Avenue, Eveleigh, New South Wales 2015, Australia; 4 Deakin University, Burwood Highway, Burwood, Victoria 3125, Australia; 5 Australian Drug Foundation, Level 12, 607 Bourke Street, Melbourne, Victoria 3000, Australia

**Keywords:** Alcohol drinking, Sports, Public health

## Abstract

**Background:**

Excessive alcohol consumption is responsible for considerable harm from chronic disease and injury. Within most developed countries, members of sporting clubs participate in at-risk alcohol consumption at levels above that of communities generally. There has been limited research investigating the predictors of at-risk alcohol consumption in sporting settings, particularly at the non-elite level. The purpose of this study was to examine the association between the alcohol management practices and characteristics of community football clubs and at-risk alcohol consumption by club members.

**Methods:**

A cross sectional survey of community football club management representatives and members was conducted. Logistic regression analysis (adjusting for clustering by club) was used to determine the association between the alcohol management practices (including alcohol management policy, alcohol-related sponsorship, availability of low- and non-alcoholic drinks, and alcohol-related promotions, awards and prizes) and characteristics (football code, size and location) of sporting clubs and at-risk alcohol consumption by club members.

**Results:**

Members of clubs that served alcohol to intoxicated people [OR: 2.23 (95% CI: 1.26-3.93)], conducted ‘happy hour’ promotions [OR: 2.84 (95% CI: 1.84-4.38)] or provided alcohol-only awards and prizes [OR: 1.80 (95% CI: 1.16-2.80)] were at significantly greater odds of consuming alcohol at risky levels than members of clubs that did not have such alcohol management practices. At-risk alcohol consumption was also more likely among members of clubs with less than 150 players compared with larger clubs [OR:1.45 (95% CI: 1.02-2.05)] and amongst members of particular football codes.

**Conclusions:**

The findings of this study suggest a need and opportunity for the implementation of alcohol harm reduction strategies targeting specific alcohol management practices at community football clubs.

## Background

Excessive alcohol consumption continues to be a primary cause of chronic and acute harm in almost all countries [1]. Causally linked to more than 60 types of injury and chronic disease, alcohol causes 3.2% of deaths worldwide and is responsible for 4.0% of disability adjusted life years [2,3]. The economic costs of alcohol abuse are estimated to be significant; for instance, in the United States the cost of alcohol abuse is predicted to be 2.7% of the country’s gross domestic product (purchasing power parity) [3].

A number of studies have demonstrated a link between sport, excessive alcohol consumption and alcohol-related harm among sports fans/spectators [4-6] and elite athletes [7]. A relationship between alcohol-related harm and sport has also been established among non-elite players and spectators of community-level sport. For example, studies conducted in England and New Zealand have reported higher levels of harmful alcohol consumption among people involved in community sporting clubs than amongst community members generally [8,9]. A number of Australian studies have also reported alcohol consumption levels among members of community sports clubs to be markedly higher than those in the general community [10,11]. One such study found that 48% of players and members of non-elite community football and cricket clubs consumed more than four drinks on a single occasion at least once a month at their sports club [11]. A similarly high prevalence of risky alcohol consumption (54% consuming six or more drinks at least once a week) has been documented amongst non-elite Gaelic footballers in Ireland [12]. Explanations as to why excessive alcohol consumption and alcohol-related harm are more prevalent amongst people involved with sport include: the ritualism associated with sporting events, with overindulgence more acceptable and even expected [4]; alcohol marketing and promotion specifically targeting sports [5]; drinking as a reward for sports participation [9]; and drinking as a coping mechanism for dealing with the stresses of sports participation [9].

In order to address such harms, the World Health Organisation’s Global Strategy to Reduce the Harmful Use of Alcohol has identified community organisations such as sporting clubs as important settings for policy interventions to reduce alcohol-related harm [13]. Governments and peak sports organisations within Australia have made similar recommendations regarding the implementation of strategies to reduce the risk of alcohol-related harms in sports clubs [14-17]. Identifying the determinants of alcohol-related harm in sporting clubs is an important first step in the development of evidence based harm reduction interventions in this setting. Research conducted in other, somewhat analogous, venues that sell alcohol (e.g. bars, taverns and pubs) suggests a range of alcohol management practices are associated with at-risk alcohol consumption and alcohol-related violence. Such practices include: possession of an appropriate license to sell alcohol; existence of an alcohol management policy; staff training in responsible service of alcohol and patron aggression management; effective supervision of alcohol-service staff [18,19]; responsible service of alcohol [19-21]; alcohol sale promotions and drinking games; and acceptance of sponsorship benefits from the alcohol industry [19,21-23].

The prevalence of alcohol management practices and the extent to which such practices are associated with alcohol-related harms and at-risk alcohol consumption in community sporting clubs is largely unreported. A literature search by the authors identified just one study that examined the association between the alcohol management practices of community (non-elite) sports clubs and alcohol-related harms or consumption [23]^a^. The study conducted in New Zealand, found that alcohol sponsorship at the individual, team and club level, particularly in the form of free or discounted alcohol, was associated with higher scores on the Alcohol Use Disorders Identification Test among players [23]. The study did not examine the relationship between other alcohol management practices of clubs and alcohol consumption. An additional study investigating the correlates of high risk alcohol consumption in Australian Rules Players was found, however, this study only included professional players [7]. This study found that players who received a drink card entitling them to free drinks were 1.68 (95% CI, 1.11 to 2.55) times more likely to report monthly risky drinking than those who did not report receiving a drink card [7].

Given the limited evidence available regarding practices that contribute to excessive alcohol consumption in community sports clubs, a study was undertaken to examine the association between the alcohol management practices and characteristics of community football clubs and at-risk alcohol consumption by club members (e.g. players, committee members, spectators and coaches).

## Methods

### Ethics approval

The study was approved by the University of Newcastle Human Research Ethics Committee on the 29/1/09 and conforms to the provisions of the Declaration of Helsinki.

### Design and setting

A cross sectional survey of community football club management representatives and members was conducted in the state of New South Wales, Australia, as part of a larger randomised controlled intervention study. The larger study assessed the effect of a two-and-a-half-year alcohol management intervention on at-risk alcohol consumption and alcohol-related harms amongst members of community sports clubs [24]. The study area included metropolitan, regional and rural communities and accounted for approximately 75% of the state population and 25% of Australia’s overall population [25].

### Sample

a) Community football clubs

The sample of clubs consisted of community-level, non-elite football clubs across four major football codes: soccer/association football, Rugby League, Rugby Union, and Australian Rules football. All are team-based ball sports predominantly involving male players, played at both the amateur and professional levels in Australia and in the first three cases internationally. Clubs were defined as amateur or non-elite if they were not a part of a major national or state level league or competition.

To ensure relevant clubs participated in the study, clubs were considered eligible if they: had players over the legal drinking age (18 years of age and over); were a non-elite community sporting club; had over 40 members (enough to participate in the survey); and, held a liquor licence enabling sale of alcohol at the sporting club.

b) Club members

Club members were eligible to participate in the study if they were 18 years of age or over and were current members of the club. Members included players, committee members, spectators and coaches.

### Recruitment procedures

a) Community football clubs

In the absence of a state or national register of community sporting clubs, a list of all community football clubs in the study area was created by contacting local councils and the peak association for each football code, and searching telephone directories and sporting web sites.

b) Club management representative

The presidents of identified clubs were invited to complete a survey on behalf of club management. Alternatively, the president was able to nominate another member of club management (eg vice president or secretary) to complete the survey for the club.

c) Club members

Lists of club members were provided by participating clubs. A quasi-random procedure was used to select club members to participate in the survey, with the 25 club members who most recently celebrated a birthday being invited to participate [26,27].

### Data collection procedures

Computer-assisted telephone surveys [28] were conducted with the nominated management representative from each of the participating clubs to assess the club’s alcohol management policies and practices (average length: 40 minutes), and with the selected members from each participating club to assess alcohol consumption at the club (average length: 19 minutes). Interviews were conducted by trained interviewers and were conducted during playing season (May to September 2009). Alcohol consumption questions were developed based on validated measures of alcohol consumption [29-31]. Alcohol management items were developed by an expert advisory group of health promotion practitioners and alcohol researchers, with reference to the scientific literature [7,32,33].

### Measures

a) Club alcohol management practices

Club representatives were asked to report on the following club alcohol management practices: liquor licence status (yes/no) and type [34]; existence of a written alcohol management policy (yes/no); alcohol-related sponsorship of the club (yes/no); sponsorship through free/discounted alcohol (yes/no); proportion of staff trained in responsible service of alcohol (all/most/some/none); how often staff consumed alcohol on duty (never/rarely/sometimes/usually/always); availability of non-alcoholic and low-alcoholic drinks (yes/no); relative pricing of low-alcohol and full-strength alcohol drinks (low-alcohol more expensive/priced the same/full-strength alcohol more expensive) [32]; availability of substantial food when alcohol is sold (snacks/light meals/full meals); and, existence of alcohol promotions: drinks discounted for a defined period of time (‘happy hour’ promotions), ‘all you can drink’ promotions, other discounted/cheap drink promotions (eg. two drinks for the price of one) [33], alcohol awards/prizes and drinking vouchers (all yes/no) [7].

b) Club code, size and location

Club representatives were also asked in the telephone interview to describe their club in terms of: football code; number of registered players (as a measure of club size); and postcode of the club’s sports ground. Postcode was used to categorise clubs as ‘major city’, ‘inner regional’ or ‘outer regional’ [35].

c) Club member alcohol consumption

Level of alcohol consumption of club members whilst at their club was assessed using a modified version of the graduated frequency index (GFI), a validated measure widely used in population surveys [29-31]. Members were asked how often they consumed the following number of standard drinks of alcohol in one drinking session at their club over the past three months: 20 or more; 11-19; 7-10; 5-6; 3-4; and 1-2 (5 to 6 days a week; 3 to 4 days a week; 1 to 2 days a week; 2 to 3 days a month; about 1 day a month; less often; or never). The GFI was modified to only cover alcohol consumed within the sports club setting and only alcohol consumed over the past three months, as this was the period when clubs were operating (sporting season). Based on Australia’s national drinking guidelines, consumption of five or more drinks at least once a month was defined as placing members at risk of immediate harm [36].

Club members were also asked how often they had witnessed alcohol being served to intoxicated people at the club and how often they had witnessed intoxicated people being admitted to the club (never/rarely/sometimes/frequently/always).

Club members were asked to report their age, gender, income and highest level of educational attainment [29].

### Statistical analyses

The following categories were used for analysis: number of players grouped into ‘less than 150’ or ‘equal to or above 150’; postcodes used to group clubs as ‘major city’, ‘inner regional’ or ‘outer regional’ [37]; proportion of staff trained in responsible service of alcohol grouped as ‘all’ or ‘most/some/none’; how often staff are allowed to consume alcohol on duty grouped as: ‘never’ or ‘rarely/sometimes/usually/always’; pricing of low-alcohol drinks relative to full-strength alcohol drinks grouped as ‘full strength drinks most expensive’ or ‘low-alcohol drinks most expensive/priced the same’; and, availability of food when alcohol sold grouped as ‘light meals/full meals’ or ‘snacks’.

Responses to questions from the graduated frequency index were used to categorised club members as consuming either ‘five or more drinks at least once a month’ or ‘not’ [38].

Analysis of the association between club alcohol management practices and characteristics, and club member alcohol consumption was undertaken as a two-step process. First, univariate analysis (chi square) was undertaken to test associations between at-risk alcohol consumption of members and 18 club alcohol management practices and three club characteristics. Second, variables with a chi-square p-value ≤0.2 were included in a backwards stepwise logistic regression analysis, controlling for age and gender as known variables associated with at-risk consumption of alcohol [36,39,40]. Variables that had a p-value greater than 0.05 were removed from the analysis one at a time until all variables in the model had a p-value below this level. Club members who reported that they abstained from consuming alcohol were excluded from such analyses.

In both stages of analysis, adjustments were made for clustering at the club-level. SAS version 9.2 was used for all analyses.

## Results and discussion

### Sample

a) Club management representatives

A total of 328 community football clubs within the study area were identified and contacted. Upon screening, 228 (70%) of these clubs were deemed eligible to participate and invited to take part in the study. Of these, 72 (32%) clubs consented to participate. Consenting clubs did not differ significantly from non-consenting clubs in terms of football code (*χ*2=6.68 df=3; p=0.0764) or location (major city; inner regional; outer regional) (*χ*2=0.20 df=1; p =0.6559). Half of the club management representatives who completed the club survey were club presidents or vice presidents (n=36; 50%), 31% were club secretaries (n=22) and 19% had other executive roles on club committees (n=14).

b) Club members

Participating clubs provided contact details for 1726 club members. Of these, 1671 (97%) were eligible to participate in the study, 1514 (90%) were able to be contacted and 1428 completed the survey - an overall consent rate of 94% and response rate of 85%. An average of 20 members per club completed the survey (range: 12–24 members). Of the 1428 club members surveyed, 7% (n=93) reported that they did not ever consume alcohol. As shown in Table 1, the vast majority of the 1335 survey participants who reported consuming alcohol were male (83%) and employed (87%) and just over half were players (55%). The average age of participants was 34 years. Study participants were comparable to participants in football codes across Australia generally in terms of gender (national data: male 82%) [41], and slightly older in terms of age (national data: average age 18–24 years) [42-45]. While equivalent national data are not available for the other variables, national data for all sports indicates that the study sample may have had more employed people (national data for all sports: 65% employed), and more people in non-playing roles (national data for all sports: 15%) [46] than the national average.

**Table 1:** Characteristics of participating club members (not including abstainers) (N=1335)

**Characteristic**	**% (number)**
*Role in club*	
Player	55% (729)
Coach	15% (197)
Club committee member	14% (193)
Club supporter/fan	11% (145)
Multiple roles	5% (71)
*Gender*	
Male	83% (1111)
Female	17% (224)
*Employment status*	
Employed	87% (1159)
Unemployed/retired/other	13% (176)
*Age*	
Mean (SD)	34 years (12 years)

Of the 1335 club members who consumed alcohol, 26% (n=366) reported drinking five or more standard drinks at the club at least once a month.

### Association between risky alcohol consumption and club alcohol management practices and characteristics

Table 2 displays the results of univariate analyses of the association between club alcohol management practices and characteristics and at-risk alcohol consumption by club members. The eight club alcohol management practices and two club characteristics with a chi square p-value of ≤0.2 were included in the subsequent logistic regression analysis.

**Table 2:** Univariate association between club alcohol management practices and characteristics and at-risk alcohol consumption by club members

**Variable**	**Members who consumed ‘5 or more drinks’ at least once a month at the club n (%)**	**p-value**
*Hold a liquor licence*		p=0.411
Yes	300 (28.4%)	
No	66 (23.8%)	
*Type of liquor licence*		p=0.572
Limited licence-single function	6 (40.0%)	
Limited licence- multiple function	242 (28.2%)	
Function licence	12 (17.6%)	
*Written alcohol management policy*		p=0.843
Yes	140 (26.9%)	
No	222 (27.9%)	
*Sponsors who make, distribute or sell alcohol*		p=0.382
Yes	316 (28.0%)	
No	50 (24.2%)	
*Alcohol received from sponsors*		p=0.248
Yes	67 (34.0%)	
No	299 (26.3%)	
*Staff trained in responsible service of alcohol*	231 (27.8%)	p=0.783
All staff trained	130 (26.5%)	
Less than all staff trained		
*Staff consumption of alcohol while on duty*		p=0.027
Never	183 (23.6%)	
Rarely/sometimes/usually/always	183 (32.7%)	
*Availability of low-alcohol options*		p=0.008
Yes	341 (26.7%)	
No	25 (42.4%)	
*Pricing of full strength and low alcohol drinks*		p=0.758
Full strength most expensive	258 (26.4%)	
Priced the same or low alcohol most expensive	69 (28.2%)	
*Availability of food when alcohol served*		p=0.947
Yes	360 (27.4%)	
No	6 (27.3%)	
*Allow intoxicated people to enter club*		p<0.001
Yes	298 (31.8%)	
No	68 (17.1%)	
*Service of alcohol to intoxicated people*		p<0.001
Yes	321 (31.7%)	
No	45 (13.9%)	
*Discounted drinks for a defined period of time*		p<0.001
*(‘Happy hour’ promotions)*		
Yes	17 (43.6%)	
No	345 (27.0%)	
*Other discounted/cheap drink promotions*		p=0.374
Yes	17 (34.7%)	
No	345 (27.2%)	
*Drinking competitions*		p=0.003
Yes	98 (40.0%)	
No	264 (24.6%)	
*All you can drink promotions*		p=0.071
Yes	37 (38.1%)	
No	325 (26.6%)	
*Alcohol-only awards or prizes*		p=0.016
Yes	108 (37.5%)	
No	254 (24.7%)	
*Vouchers for free alcoholic drinks*		p=0.216
Yes	51 (34.5%)	
No	311 (26.6%)	
*Football code*		p=0.001
Soccer/association football	55 (17.9%)	
Rugby League	103 (25.1%)	
Australian Rules	58 (25.8%)	
Rugby Union	150 (38.2%)	
*Number of players*		p=0.008
Less than 150	181 (34.0%)	
150 or more	185 (23.0%)	
*Geographical location*		p=0.288
Major cities	306 (27.3%)	
Inner regional	27 (20.0%)	
Outer regional	33 (40.7%)	

As shown in Table 3, five of the 10 variables entered into the logistic regression model were independently associated with members consuming alcohol at levels linked with immediate harm (p<0.05).

**Table 3:** Multivariate association between club alcohol management practices and characteristics and at-risk alcohol consumption by club members

**Variable**	Adjusted odds ratio (95% CI)*	**p value**
*Service of alcohol to intoxicated people*		p=0.0074
No	Referent	
Yes	2.23 (1.26–3.93)	
*Happy hour promotions*		p<0.0001
No	Referent	
Yes	2.84 (1.84–4.38)	
*Alcohol.only awards or prizes*		p=0.0084
No	referent	
Yes	1.80 (1.16–2.80)	
*Football code*		p=0.0004
Australian Rules	referent	
Soccer/association football	1.25 (0.70–2.24)	
Rugby League	1.95 (1.10–3.46)	
Rugby Union	2.64 (1.60–4.37)	
*Number of players*		p=0.0393
150 or more	referent	
Less than 150	1.45 (1.02–2.05)	

* Adjusted for clustering of members at the same club.

Members of clubs where service of alcohol to intoxicated people was observed had significantly greater odds of consuming alcohol at risky levels than members of clubs were this practice was not observed (OR: 2.23). This was also the case for members of clubs that had happy hour promotions (OR: 2.84) or alcohol-only awards/prizes (OR: 1.80).

Members of Rugby Union and Rugby League clubs had significantly greater odds of consuming alcohol at risky levels compared to Australian Rules football club members. Rugby Union club members had significantly greater odds (adjusted odds ratio: 2.1; 95% CI: 1.28-3.47) of consuming alcohol at risky levels compared to soccer/association football club members. Members of clubs with less than 150 players had significantly greater odds of consuming alcohol at risky levels compared to members of larger clubs.

The findings of this study suggest that a number of modifiable alcohol management practices are associated with at-risk alcohol consumption by community sport club members, and that such consumption is more likely to occur in small clubs and in specific football codes. Service of alcohol to intoxicated people, happy hour promotions (where alcohol is provided at a discounted rate for a defined period of time) and alcohol-only awards or prizes were found to be associated with club members being more than twice as likely to consume alcohol to excess. These findings confirm the need and the opportunity for the development and implementation of alcohol harm reduction interventions in these settings [13].

To our knowledge, this is the most comprehensive study examining the association between the consumption of alcohol by club members and the alcohol management practices of sporting clubs, at either the community or professional level [7,23]. The identification of service of alcohol to intoxicated people, happy hour promotions and alcohol-only awards or prizes as predictors of at-risk alcohol consumption is consistent with work conducted in other drinking contexts [18-21], and with professional sports players [7]. For instance, Dietze and colleagues [7] found greater alcohol consumption among professional football players who received a drink card entitling them to free drinks, which is analogous to the alcohol-based awards and prizes examined in this study. Also consistent with our findings, Dietze and colleagues found that having formal club rules (or policy) on alcohol consumption was not a predictor of excessive alcohol consumption amongst professional players. Such findings are consistent with evidence from the broader body of scientific literature on licensed premises in general, which suggests that policies alone are insufficient to reduce excessive alcohol consumption and prevent alcohol-related harm [47].

This study’s finding regarding a lack of association between alcohol-related sponsorship of clubs and at-risk alcohol consumption by club members is in contrast with that of O’Brien and Kypri [23], who found that players receiving alcohol sponsorship had significantly higher scores on the Alcohol Use Disorders Identification Test than those receiving no sponsorship. A number of factors may account for this contrast. First, the O’Brien and Kypri [23] study examined an individual’s total alcohol consumption (not only that within the club setting) and covered a number of sports, whereas the present study only investigated the consumption of alcohol within the sporting club setting and focused solely on football clubs. Furthermore, O’Brien and Kypri [23] examined sponsorship at individual, team and club levels whereas the current study examined sponsorship at the club-level only. Future studies seeking to explore the impact of alcohol-related sports sponsorship may benefit from utilising the more comprehensive approach reported by O’Brien and Kypri [23].

While previous research has found a positive association between the size of licensed premises and alcohol-related violence (with larger premises associated with greater levels of violence), the relationship between premises size and level of alcohol consumption has received less attention in the literature [48]. The findings of this study, which suggest that excessive alcohol consumption is greater in smaller clubs, appear to be in contrast to that reported in relation to licensed premises size and alcohol-related violence. As sporting clubs are typically staffed by volunteers [49] and alcohol consumption is often permitted in large areas surrounding playing fields, including grandstands [50], the increased risk of at-risk alcohol use within smaller clubs may reflect a lower level of capacity of such clubs to monitor and manage alcohol consumption compared to larger clubs.

By targeting those club practices that have been identified as predictors of at-risk alcohol consumption, community sports clubs have an opportunity to reduce alcohol-related harm involving players and spectators. A number of interventions in non-sporting licensed venues have been found to reduce patron intoxication and prevent alcohol-related harm [1,18-21] and the potential exists for such interventions to be similarly effective if appropriately tailored to the sports club setting and focussed on the predictors identified in this study. For instance, such interventions should prohibit happy hour promotions and alcohol-only awards and prizes and include strategies to assist staff or volunteers to identify intoxicated patrons, refuse them service of alcohol and ask them to leave the club (actions that are consistent with current state liquor laws [34]). However, as with non-sporting licensed premises, it is unlikely that these interventions would be effective without adequate monitoring and enforcement [1,18,21,34,47,51]. Given the cost of enforcement, a lower cost strategy for increasing the uptake of harm reduction strategies by clubs is through accreditation with a recognised authority, as has been described by Duff and Munro [49]. Findings from past, non-randomised studies suggest that such an intervention approach may be acceptable [52] and have the potential to reduce at-risk alcohol consumption and related harm [53,54].

The results of this study need to be considered in the context of the study methodology. First, the study relied on the self-report of club delegates to report on club alcohol management practices. While the validity of such self-reported assessments are not known, self-reported assessment of the health promotion policies and practices of alcohol outlets as well as community organisations such as schools and childcare services is common [55-57], with representatives of such organisations having previously been found to validly and reliably report organisational policies and practices [58,59].

The low club consent rate (32%) obtained for this study is likely to be related to clubs being concurrently recruited into a large intervention trial that required an ongoing commitment to data collection and other activities for a four-year period [24]. Comparison of the characteristics of consenting and non-consenting clubs in terms of football code and location suggested that this risk of bias was limited. Finally, the study involved participation by clubs that served 75% of the population in the most populous state in Australia. The extent to which the findings are generalisable to other states, to other sports codes, and to other countries is unknown and warrants further study.

Further research should be conducted to confirm the findings of this study, particularly in the context of sports other than football. It would also be beneficial if future research investigated the relationship between, and relative importance of, individual and club-based predictors of excessive alcohol consumption and alcohol-related harm.

## Conclusions

This study highlights the importance of both modifiable practices and club characteristics as factors associated with at-risk alcohol consumption at community football clubs. Given the extensive participation of community members in football clubs around the world, an opportunity exists to reduce alcohol-related harm associated with at-risk consumption by club players and spectators through the implementation of appropriately tailored and targeted alcohol management strategies.

## Endnote

^a^The authors search the following databases: The Cochrane Library (1996–2013), MEDLINE (1950–2013), EMBASE (1988–2013), PsychINFO (1806–2013), CINAHL (1956–2010) and SPORTDiscus (1977–2013), using the search terms ‘alcohol’ and ‘sport’. Articles were eligible for inclusion in the review if they examined club-based predictors of excessive alcohol consumption or alcohol-related harm amongst amateur or non-elite athletes/other club members.

## Competing interests

The authors declare that they have no competing interests.

## Authors’ contributions

MK drafted the manuscript and conducted a review of the literature and the statistical analysis. JW and LW helped to conceive the study, participated in its design and helped to draft the manuscript. BR and SW assisted with the literature review and BR assisted with the statistical analysis. KG, VK, RR and RC provided conceptual advice and advice regarding the setting and participants. All authors read and approved the final manuscript.

## Pre-publication history

The pre-publication history for this paper can be accessed here:

http://www.biomedcentral.com/1471-2458/13/762/prepub
